# Audit of a program to increase the use of vacuum extraction in Mulago Hospital, Uganda

**DOI:** 10.1186/s12884-016-1052-3

**Published:** 2016-09-02

**Authors:** Barbara Nolens, John Lule, Flavia Namiiro, Jos van Roosmalen, Josaphat Byamugisha

**Affiliations:** 1 Department of Obstetrics and Gynaecology, Mulago National Referral Hospital, PO Box 7051, Kampala, Uganda; 2 Department of Obstetrics and Gynaecology, Canisius-Wilhelmina Hospital, PO Box 9015, 6500GS Nijmegen, The Netherlands; 3 Athena Institute, VU University, PO Box 22700, 1100DE Amsterdam, The Netherlands; 4 Makerere University, School of Medicine, College of Health Sciences, PO Box 7072, Kampala, Uganda; 5 Department of Paediatrics, Mulago National Referral Hospital, PO Box 7051, Kampala, Uganda

**Keywords:** Vacuum extraction, Implementation, Perinatal outcome, Stillbirth, Neonatal death, Uterine rupture, Low-income country, Audit

## Abstract

**Background:**

Prolonged second stage of labour is a major cause of perinatal and maternal morbidity and mortality in low-income countries. Vacuum extraction is a proven effective intervention, hardly used in Africa. Many authors and organisations recommend (re)introduction of vacuum extraction, but successful implementation has not been reported. In 2012, a program to increase the use of vacuum extraction was implemented in Mulago Hospital, Uganda. The program consisted of development of a vacuum extraction guideline, supply of equipment and training of staff. The objective of this study was to investigate the impact of the program.

**Methods:**

Audit of a quality improvement intervention with before and after measurement of outcome parameters. Setting: Mulago Hospital, the national referral hospital for Uganda with approximately 33 000 deliveries per year. It is the university teaching hospital for Makerere University and most of the countries doctors and midwives are trained here. Data was collected from hospital registers and medical files for a period of two years. Main outcome measures were vacuum extraction rate, intrapartum stillbirth, neonatal death, uterine rupture, maternal death and decision to delivery interval.

**Results:**

Mode of delivery and outcome of 12 143 deliveries before and 34 894 deliveries after implementation of the program were analysed. The vacuum extraction rate increased from 0.6 – 2.4 % of deliveries (*p* < 0.01) and was still rising after 18 months. There was a decline in intrapartum stillbirths from 34 to 26 per 1000 births (-23.6 %, *p* < 0.01) and women with uterine rupture from 1.1 – 0.8 per 100 births (-25.5 %, *p* < 0.01). Decision to delivery interval for vacuum extraction was four hours shorter than for caesarean section.

**Conclusions:**

A program to increase the use of vacuum extraction was successful in a high-volume university hospital in sub-Saharan Africa. The use of vacuum extraction increased. An association with improved maternal and perinatal outcome is strongly suggested. We recommend broad implementation of vacuum extraction, whereby university hospitals like Mulago Hospital can play an important role.To support implementation, we recommend further research into outcome of vacuum extraction and into vacuum extraction devices for low-income countries. Such studies are now in progress at Mulago Hospital.

## Background

With 293 000 maternal deaths and 5.3 million stillbirths and neonatal deaths per year, global maternal and perinatal mortality rates have decreased since 1990, but far below targets and the numbers are still alarming [1–3]. Worldwide approximately 800 women and 14 500 babies die every day because of complications of pregnancy and childbirth. Intrapartum complications are responsible for more than one third of these deaths [1–3]. Many complications are preventable or treatable with known evidence-based interventions [2, 4–6]. An important cause of maternal and perinatal morbidity and mortality is prolonged second stage of labour and its complications such as haemorrhage, sepsis, uterine rupture, obstetric fistula and birth asphyxia [1, 5, 7]. Vacuum extraction is one of the evidence-based interventions that can prevent complications by shortening the second stage of labour [8–11]. It also prevents women from having a caesarean section with its increased risk of maternal and perinatal morbidity and mortality in the index and subsequent pregnancies compared to (assisted) vaginal delivery [12–15]. Use of vacuum extraction varies widely between countries and hospitals. In 31 European countries, rates of instrumental vaginal delivery varied between 0.5 and 16.4 % [16]. In the Netherlands 9 %, in the UK 6 % and in the US 3 % of deliveries are by vacuum extraction [17–19]. While some decades ago vacuum extraction was still widely practiced in low-income countries (LIC), nowadays it is hardly used, with some exceptions [20–27]. Many authors and organizations, including the World Health Organization, recommend the use of vacuum extraction [4, 5, 20, 21, 28–30]. But successful implementation has not been reported. Reasons mentioned for the infrequent use of vacuum extraction are lack of skilled operators, equipment and training opportunities and beliefs of health care providers concerning trauma to the baby and HIV-transmission [20–22, 24, 28]. Fear of litigation and financial incentives may also play a role [16]. In 2012, a program to increase the use of vacuum extraction was implemented in Mulago Hospital, Uganda. The program consisted of development of a vacuum extraction guideline, supply of equipment and training of staff. The objective of this study was to investigate the impact of the program.

## Methods

The study design is audit of a quality improvement intervention with before and after measurement of outcome parameters. The setting is Mulago Hospital in Kampala. This is the national referral hospital in Uganda and the university teaching hospital for Makerere University. It is Uganda’s main training facility for doctors and midwives. Every year 100 midwives, 140 doctors and 20 specialists in obstetrics & gynaecology graduate here. With approximately 33 000 deliveries per year, it has one of the busiest maternity units in the world. The study was performed in the labour ward for women with medium to high-risk pregnancies, where maternity services are free of charge. Every month approximately 2000 women deliver in this ward, many of them after referral because of complications. Women come mainly from Kampala and surroundings, but some have to travel for a day to reach this hospital. There is an obstetric high-care unit where care is given to women with severe complications, such as uterine rupture, severe haemorrhage, sepsis and eclampsia. There is a neonatology unit where care is given to babies with severe morbidity, such as prematurity and birth asphyxia.

Together with Mulago Hospital’s obstetricians and the Hospital Hygiene department, standard operating procedures (SOP) for the use of vacuum extraction and sterilization of Kiwi vacuum extractors (Clinical Innovations, USA) were developed [31, 32]. Used Kiwi vacuum extractors (type OmniCup) were donated by several hospitals in the Netherlands and sterilized according to the SOP. Sterilization was repeated after every use. Training took place in the hospital. All 45 residents (in training to become specialists in obstetrics & gynaecology) were trained in small groups of four to six doctors in the week before they had a duty-week on labour ward. Training was provided by the first author and consisted of discussion of the SOP on vacuum extraction and sterilization, watching the World Health Organization Reproductive Health Library video on vacuum extraction and skills training on mannequins [33]. They had on the job supervision in the week after the training. After completion of this program with a duration of 4 months, training continued according to the existing curriculum complemented with the new SOP on vacuum extraction. It consisted of a yearly theory- and a yearly skills training session per year group for all residents and medical students in their last year, provided by Mulago Hospital’s specialists (six theory and six skills training sessions during 18 months follow up). Data was collected for a baseline period of six months before- and a follow up period of 18 months after implementation. The follow up period started at the time of implementation. To investigate uptake and success rate of vacuum extraction, information on the following outcome measures was collected from the registers of labour ward and the obstetric operating theatre: successful vacuum extraction and failed vacuum extraction. Successful vacuum extraction was defined as delivery by vacuum extraction, irrespective of maternal or perinatal complications. Failed vacuum extraction was defined as an attempted vacuum extraction whereby the procedure was abandoned, usually because the stopping criteria were met. Stopping criteria were: the baby’s head is not delivered or about to be delivered after three traction-aided contractions; the vacuum pops off three times or 20 min have passed after application of the cup [31]. To investigate the impact on perinatal outcome, information on the following outcome measures was collected for all deliveries on the medium to high-risk labour ward during the study period: intrapartum stillbirth, macerated stillbirth, neonatal death with birthweight of ≥ 2.5 kg, admission to the neonatology unit with birthweight of ≥ 2.5 kg and total perinatal deaths. In Mulago Hospital the gestational age is often not known. We used low birthweight (< 2.5 kg) as a proxy for preterm birth. Outcome for babies with birthweight ≥ 2.5 kg was investigated separately, because most vacuum extractions are done in this group. Total perinatal deaths was defined as all stillbirths plus all neonatal deaths during admission. This included low birthweight stillbirths and low birthweight neonatal deaths.

To measure maternal outcome, information on the following outcome measures was collected: uterine rupture, admission to obstetric high-care unit and maternal death. Data were obtained from the records department and the registers of the labour ward, obstetric high-care unit, obstetric operating theatre and neonatology unit. In addition to this, medical files of those women who had vacuum extraction during the last six months of the study were investigated for maternal and perinatal outcome and decision to delivery interval (DDI). DDI was defined as time between doctors’ decision to do a vacuum extraction (as noted in file) and time of birth. Data was entered into MS Excel 2013 and imported into Statistical Package of the Social Sciences (SPSS) 22.0 for analysis. Observations before and after implementation of the program were compared. Results are reported in numbers and proportions. The chi-square test was used for comparison of the categorical variables. *P*-values <0.05 were considered statistically significant.

Ethical permission to conduct this study was obtained from the Mulago Hospital Research and Ethics Committee (refnr: MREC 489) and the Uganda National Council for Science and Technology (refnr: HS1752).

## Results

### Overall outcome

**Fig. 1:**
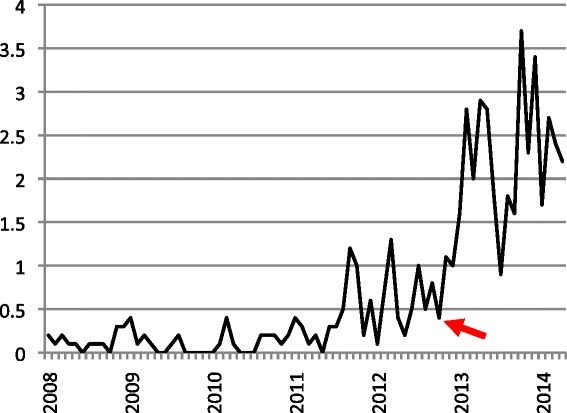
Monthly vacuum extractions as percentage of all deliveries, January 2008 - April 2014. *Arrow*: start of program in November 2012

**Fig. 2:**
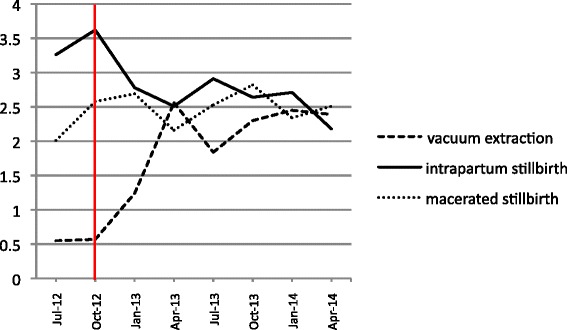
Vacuum extractions, intrapartum stillbirths and macerated stillbirths as percentage of all deliveries. Percentages are calculated per period of three months. *Vertical line*: start of the program

During the two-year study period from May 2012 to May 2014, 47 037 deliveries were registered on the medium to high-risk labour ward: 12 143 in the baseline period and 34 894 in the follow up period. The use of vacuum extraction increased from 0.6 % to a maximum of 3.7 % and stabilized at 2.4 % of all deliveries on this ward (Figs. 1 and 2). In the first six months after implementation vacuum extraction was used in 1.9 %, in the next six months 2.1 % and the last six months 2.4 % of deliveries. The vacuum extraction rate in the total follow up period was 2.1 % (Table 1). In the 18 months after implementation 805 vacuum extractions were performed with 63 failures (8.5 %).

**Table 1:** Maternal and perinatal outcome in Mulago Hospital medium to high-risk labour ward in baseline and follow up period

	Baseline		Follow up		Impact	*p*-value
Duration	6 months		18 months			
Total deliveries	12143		34894			
	*n*	(%)	*n*	(%)		
Vacuum extraction	68	(0.6)	742	(2.1)	+280 %	<0.01
Caesarean section	3427	(28.2)	10550	(30.2)	+7.1 %	<0.01
Caesarean section for obstructed labour	729	(6.0)	2106	(6.0)	0 %	0.9
Maternal outcome						
Ruptured uterus	133	(1.1)	287	(0.8)	-25.5 %	<0.01
Admissions to obstetric high-care unit^a^	228	(1.9)	629	(1.8)	-4.3 %	0.59
Maternal deaths	*n*	(per 100 000)	*n*	(per 100 000)		
Maternal death due to intrapartum complication	32	(264)	82	(235)	-11.0 %	0.58
Maternal death due to hypertensive disorder	13	(107)	48	(138)	+29.0 %	0.42
Maternal death due to abortion	22	(181)	65	(186)	+2.8 %	0.91
All maternal deaths	97	(799)	254	(728)	-8.8 %	0.43
Perinatal outcome	*n*	(‰)	*n*	(‰)		
Total perinatal death	1112	(91)	2946	(84)	-7.7 %	0.02
Macerated stillbirth	278	(23)	877	(25)	+9.6 %	0.17
Intrapartum stillbirth	417	(34)	914	(26)	-23.6 %	<0.01
"Term" neonatal death	156	(13)	482	(14)	+7.8 %	0.43
"Term" admissions to neonatology unit	1060	(87)	3482	(100)	+14.4 %	<0.01

Definitions used in this study:

Total perinatal death: stillbirths + neonatal deaths during admission

Stillbirth: baby born with no signs of life at or after 28 weeks gestation or with a birthweight of 1 kg or more

Neonatal death: death during admission after live birth "term": birthweight of 2.5 kg or more

^a^For intrapartum complication

Total perinatal mortality decreased from 91 per 1000 births in the baseline period to 84 per 1000 births in the follow up period (*p* < 0.05). This was mainly a result of a decrease in intrapartum stillbirths from 34 per 1000 to 26 per 1000 births, a decrease of 23.6 % (*p* < 0.01). Admission of term babies to the neonatology unit, however, increased with 14.4 % (*p* < 0.01) from 87 – 100 per 1000 births (Table 1, Fig. 2). Decrease in intrapartum stillbirths was most notable in the last six months of the study with 24 per 1000 births, a decrease of 28.7 %, when the vacuum extraction rate was at its highest (Fig. 2). The macerated stillbirth rate did not change (Fig. 2).

Maternal deaths from intrapartum complications, such as haemorrhage, sepsis, uterine rupture and obstructed labour showed a downward trend from 264 to 235 per 100 000 births (-11.0 %), but this did not reach statistical significance. Admissions to the obstetric high-care unit for intrapartum complications showed a downward trend as well. The number of women with ruptured uterus decreased by 25.5 % (*p* < 0.01). Maternal deaths from abortions and hypertensive disorders remained the same or increased (Table 1).

### Outcome of vacuum extraction

During the last six months of the study, 342 vacuum extractions were attempted of which 32 failed (9.4 %). Mean DDI for (attempted) vacuum extraction was 34 min. After exclusion of 15 women with intra uterine foetal death before vacuum extraction and one woman with unknown outcome, perinatal outcome of 326 (attempted) vacuum deliveries could be analysed, 296 vacuum extractions and 30 failed vacuum extractions. The perinatal mortality rate was 19/326 (58 per 1000 births) for all attempted vacuum extractions with a live foetus at time of decision for intervention. It was documented in 35 % of files that emergency caesarean section was planned initially. However, while the woman was waiting for caesarean section the planned mode of delivery changed to vacuum extraction due to various reasons (different findings on examination, more experienced doctor, foetal distress, no theatre space available). Vacuum extraction was successful in 93.3 % of women initially scheduled for caesarean section, comparable to women not scheduled for caesarean section.

## Discussion

### Increased use of vacuum extraction

After implementation of a program to increase the use of vacuum extraction in Mulago Hospital Uganda, the use of this intervention rose rapidly. Within a few months it became a routine procedure that was used daily. After 18 months, at the end of the study period, more than 800 vacuum extractions had been performed and the vacuum extraction rate was still rising. This study shows that implementation is possible in a high-volume university hospital in a LIC and that vacuum extraction is accepted by health care providers. What is needed is training and equipment. This might sound straight forward and many authors and organizations advise implementation of vacuum extraction to LIC [4, 5, 20, 21, 28–30]. But to our knowledge successful projects of this size have not yet been published. A key to success might be involving major university hospitals. Our approach of incorporating the program into the medical curriculum of a national referral and university hospital where the majority of doctors and midwives for the country are trained had several benefits: The program was efficient in training many health care providers in a relatively short period of time. Many women could benefit from the procedure and trainees did get enough exposure. Doctors and midwives trained in this institution took their knowledge and skills to all parts of the country. Furthermore, senior specialists who are lecturers at the country’s major medical university and opinion leaders about medical practice in the country, were consulted and supported the program. Nevertheless, regular (in-service) training, updates and skills and drills sessions for all health care providers attending delivery in smaller health units is needed as well.

Increased use of vacuum extraction, as seen in our study, might not only lead to better maternal and perinatal outcome, but in a high fertility environment like Uganda, it could have a huge impact on future healthcare costs by reducing the number of second stage caesarean sections.

### Improved perinatal and maternal outcome

In settings where foetal monitoring is adequate and timely access to the operating theatre for caesarean section is guaranteed, increasing the vacuum extraction rate (and decreasing the caesarean section rate) would probably result in better maternal outcome but might not have a measurable effect on perinatal outcome. In LIC where access to the operating theatre is often delayed, timely delivery by vacuum extraction might have a major effect on perinatal outcome as well.

In this study we observed that, while the vacuum extraction rate increased, perinatal mortality decreased. Although this observational study cannot prove causality an association is strongly suggested. An important factor is DDI. Mean DDI for (attempted) vacuum extraction was 34 min. Mean DDI for caesarean section in the second stage of labour is four hours and 38 min in Mulago Hospital (Unpublished data from ongoing study in Mulago Hospital by the same authors). Although theatre is functioning 24 h per day, demand caused by the overwhelming number of deliveries exceeds its maximum capacity. Vacuum extraction shortens the second stage of labour in women with an indication for intervention with four hours. Foetuses that otherwise would have died from birth asphyxia during this waiting time have now probably survived. This results in a shift from intrapartum stillbirths to live births. Some of these live births however, would need admission to the neonatology unit. This might explain the increase of admissions to that unit. Perinatal mortality after (attempted) vacuum extraction on a live foetus is 58.3 per 1000 in this study. Interpretation of this outcome is difficult, because literature on outcome of vacuum extraction in sub-Saharan Africa is scarce [22, 23, 27]. Birth asphyxia is probably the major cause of perinatal death, rather than complications from the vacuum extraction procedure. More research is needed into outcome of vacuum extraction in LIC, especially because concern about trauma to the baby is often mentioned as a reason for not doing vacuum extraction (Unpublished data from ongoing study in Mulago Hospital by the same authors).

Uterine rupture is a severe complication of labour with a high risk of maternal and perinatal mortality. In LIC its prevalence ranges from 0.1 to 2.9 % of deliveries [34–36]. The number of women who sustained uterine rupture in Mulago Hospital decreased after implementation of the program. This might also be explained by the shorter DDI for vacuum extraction compared to caesarean section. The downward trends in admissions to the obstetric high care unit and maternal deaths from intrapartum complications may be a result of the shorter DDI as well. Prevention of difficult caesarean sections with a deeply impacted foetal head might have had a positive effect.

### Vacuum extraction device

During this study Kiwi vacuum extractors, designed for single use, were re-used. This is done in many hospitals in LIC but has never been published. Re-use of Kiwi vacuum extractors is done in Mulago Hospital to ensure availability of ready-to-use vacuum extractors at all times and to keep costs low. Kiwi vacuum extractors are always complete, ready to use and can be operated by one person. Because of this, the procedure can be performed quickly, without losing time looking for an assistant or missing parts. We are of the opinion that Kiwi-vacuum extractors can safely be re-used if a rigorous infection control protocol is in place. Together with the Hospital Hygiene Department we designed a SOP for sterilization of Kiwi vacuum extractors [32]. The program, including the re-use of Kiwi vacuum extractors, was approved by the Mulago Hospital Research and Ethics Committee and the Uganda National Council for Science and Technology.

We acknowledge that re-using a devise that is designed for single use is not ideal. Problems we encountered during this study were: temporarily unavailability of Cidex, so that sterilisation and re-use was not possible and problems with creating a vacuum after 3-5 times of use. On the other hand, the user-friendliness of the Kiwi vacuum extractor might have contributed to the fast uptake of the intervention. However, now that vacuum extraction is a routine intervention in Mulago Hospital, we have re-introduced other types of vacuum extractors as well (Bird and soft-cup with different types of pumps) and we are investigating what type would be the most helpful in terms of user-friendliness, patient-friendliness, safety, effectivity and costs in our setting. So far, we have not found the ideal vacuum extractor. We would recommend the development of an affordable user-friendly vacuum extractor, or making the existing Kiwi device affordable as single-use instrument for LIC.

### Failure rate

In the literature failure rates of 5.6 to 34 % are described [23, 37–39]. Although the 8.5 % failure rate in this study is in the lower range of what is described elsewhere, failed vacuum extractions are a cause for concern. If a difficult procedure is expected, trial of vacuum extraction in theatre with everything in place for caesarean section in case of failure is advisable.

### Limitations

A limitation of the study is the design with before and after measurements. Although it seems plausible, it cannot prove that the increased vacuum extraction rate has caused better maternal and perinatal outcome. Randomization was not considered ethical, because vacuum extraction was not new to Mulago Hospital and because vacuum extraction is a known effective intervention elsewhere. Randomization in the setting of Mulago Hospital would mean that half of the women would have to wait for an extra four hours for caesarean section. During this waiting time they would be at risk of developing uterine rupture and/or intrapartum stillbirth. They would have a high risk operation and a uterine scar with an increased risk of complications in next pregnancies, while a vacuum extraction would have been possible there and then.

During the study period there was no other ongoing intervention in Mulago Hospital that may have accounted for the observed outcome.

## Conclusions

A program to increase the use of vacuum extraction was successful in a high-volume university hospital in sub-Saharan Africa. The use of vacuum extraction increased. An association with improved maternal and perinatal outcome is strongly suggested. The much shorter decision to delivery interval for vacuum extraction compared to caesarean section probably plays an important role. We recommend broad implementation of vacuum extraction, whereby university hospitals like Mulago Hospital can play an important role. To support implementation, we recommend further research into (long term) outcome of vacuum extraction and into vacuum extraction devices for low-income countries. Such studies are now in progress at Mulago Hospital.

## Abbreviations

DDI: Decision to delivery interval
LIC: Low-income countries
SOP: Standard operating procedure
